# Healthcare professionals’ perspectives of pharmacist roles in residential aged care: a qualitative systematic review and meta-synthesis

**DOI:** 10.1007/s11096-026-02166-4

**Published:** 2026-05-28

**Authors:** Tiernan McDonough, Emily Griffin, Amy Page, Lisa Kalisch Ellett, Christopher Etherton-Beer, Jacinta Johnson

**Affiliations:** 1 School of Pharmacy and Biomedical Science, College of Health, Adelaide University, Level 6, Bradley Building, Adelaide, SA 5000 Australia; 2 University of Western Australia, Perth, WA 6009 Australia; 3 Monash Rural Health, Monash University, Bendigo, 3550 Australia

**Keywords:** Health personnel, Homes for the aged, Interprofessional relations, Nursing homes, Professional role

## Abstract

**Introduction:**

Aged care systems are under increasing pressures, demanding optimised interdisciplinary teams. Pharmacist roles are expanding into these teams, and successful integration requires an understanding of team member perspectives.

**Aim:**

This systematic review and meta-synthesis aimed to identify, analyse and present the published literature pertaining to healthcare professional perspectives of the roles of pharmacists working in residential aged care settings.

**Method:**

A systematic search of literature published between 2000 and 2025, in English language only, was undertaken across Embase, Medline, CINAHL and Web of Science. Primary studies addressing the research aim were eligible for inclusion. The Mixed Methods Appraisal Tool was used to assess methodological quality of each paper; no papers were excluded based on quality. Two researchers independently reviewed and reached consensus agreement for all studies to include. Both researchers undertook a thematic synthesis of qualitative data to identify analytic themes.

**Results:**

After removing duplicates, 1874 unique papers were identified through database searching and an additional two papers identified through citation searching. After screening, we included 39 papers for data extraction and analysis. Three overarching themes were identified. Theme 1: ‘Supporting the role’ describes how pharmacist roles in aged care are supported through building trust with the team, education and experience, access to information, specific attributes, organisational buy-in, favourable models of care, and role clarity. Theme 2: ‘Medicines expertise activities’ describes how pharmacists perform three key roles valued by healthcare staff: knowledge and communication brokers, filling existing gaps in care, and optimising quality use of medicines. Theme 3: ‘Helping the team’ illustrates health professionals’ perception of three distinct outcomes of pharmacist input (ie enhanced confidence, improved workforce capacity and capability, and improved person-centred care).

**Conclusion:**

This meta-synthesis of the evidence regarding the perceptions of healthcare professionals on the role of pharmacists in aged care provides contextual information for individuals, organisations and policy-makers for future implementation. Pharmacists are perceived to improve stakeholder confidence, staff capacity and capability, and overall person-centred care. Embedded roles that foster interdisciplinary collaboration are preferred to irregular visiting roles. These embedded roles are enabled through a range of mechanisms that policymakers, organisations and individuals may leverage for successful implementation in future iterations.

**Supplementary Information:**

The online version contains supplementary material available at 10.1007/s11096-026-02166-4.

## Impact statement

- Synthesis of current evidence suggests that valuable pharmacist role categories involve communication and knowledge brokers, filling existing gaps in care, and optimising quality use of medicines on individual and facility levels.
- Healthcare professionals believe that pharmacists enhance confidence of staff and residents, capacity and capability of the workforce, and overall person-centred care.
- Successful roles are facilitated through building trust with the team, in-person collaboration, access to resources, education and experience of pharmacists, particular attributes of pharmacists, supportive organisations, and clear roles.

## Introduction

Aged care health systems internationally are under increasing pressures due to economic and resource challenges, health impacts of the climate crisis, and an ageing population [1–9]. As models of care move to support older adults to live at home for longer, the population entering Residential Aged Care Homes (RACHs) is increasingly frail, exposed to multimorbidity and polypharmacy. Consequently, this population experiences increased medicines-related risks such as unplanned hospitalisations, falls, and cognitive impairment. Well-implemented interdisciplinary care interventions for older persons are associated with improved patient safety and satisfaction, clinical outcomes, and resource utilisation [10–15].

Pharmacist roles are evolving to complement existing healthcare teams and models of care to reduce medicines-related harms, improve continuity of care, and reduce healthcare expenditure [16–20]. This is important in the care of older persons, where medicines-related harms are consistently described across the literature and previous research has identified benefits of pharmacists in enhancing medication safety, particularly through decreasing polypharmacy and psychotropic exposure [17, 21–24].

Interdisciplinary care can reduce healthcare utilisation, improve practitioner performance, and enhance health outcomes for individuals [25, 26]. In countries such as the United Kingdom, Australia, and the United States, pharmacists are working to achieve this through integration into interdisciplinary teams within RACHs [17, 27, 28]. Pharmacists are key members of this interdisciplinary team, providing expertise in medication management, education, and person-centred decision-making [29].

Success in team-based healthcare requires clear and respected roles, shared goals, and successful communication strategies [14, 15, 30]. Previous research has measured the outcomes, types of roles, and extent of collaboration between pharmacists and other healthcare professionals in aged care [17, 31]. Understanding the perceptions of healthcare professionals around these roles by thematically synthesising the literature will provide clarity on future directions that align with stakeholder expectations and beliefs. This will provide important contextual understanding to support the ongoing integration of pharmacists into aged care teams and optimise their potential for collaborative person-centred care.

### Aim

This systematic review and meta-synthesis aimed to identify, analyse and present the published literature pertaining to healthcare professional perspectives of the roles of pharmacists working in residential aged care settings.

## Method

The systematic review protocol was registered with PROSPERO (CRD42024584994). The systematic review was conducted and a qualitative meta-synthesis employed to analyse the primarily qualitative data. The study results are reported in line with the Preferred Reporting Items for Systematic Reviews and Meta-Analyses (PRISMA) guidelines [32].

### Selection criteria

Papers were eligible for inclusion provided they included the sample of interest (healthcare professionals within or associated with residential aged care facilities, including external contracted staff). Papers were eligible for inclusion if they addressed the phenomenon of interest (i.e. the perspectives of the role of pharmacists working in the RACH). All study designs were included provided they presented original qualitative data, either alone or within mixed-method approaches.

Literature was excluded if it only considered perceptions of non-healthcare professionals, or healthcare professionals not working within RACHs. Literature reviews and conference abstracts were excluded. Grey literature was not included to prioritise published, peer-reviewed literature, and as adequate qualitative data was expected to be obtained.

### Search strategy

A systematic search of English-language primary literature from 2000 to October 2024 was conducted across Embase, Medline, CINAHL, and Web of Science, supplemented by backward and forward citation searching. The search was updated in December 2025 to capture additional papers published. Two researchers (TM and EG) independently screened all titles and abstracts, then full texts, and reached consensus agreement for all included studies using Covidence software [33].

The search strategy was developed, tested and refined to ensure sensitivity for relevant data through use of seed papers and consultation with the co-authors, all of whom are health professionals who have published literature pertaining to older persons. Search terms encompassed four parameters: pharmacists, perceptions, residential aged care, and health professionals. Truncation and controlled vocabulary were used as relevant across databases. The search strategy was adapted for individual databases, and is included in Appendix 1.

### Critical appraisal

A critical appraisal was undertaken across all included papers by TM and AP independently using the Mixed Methods Appraisal Tool [34]. Any conflicts were resolved between both authors.

### Data collection and interpretation

Study characteristics were extracted by TM and checked for accuracy by EG. Included papers were uploaded into NVivo 15 by two researchers in full for analysis (TM and EG). Both researchers undertook a meta-synthesis of the qualitative data in accordance with the guidance for thematic synthesis set out by Thomas and Harden [35]. Quantitative data were read and used to support interpretative analysis of qualitative findings; however, it was not formally analysed nor presented in this review given our interest in the qualitative findings only. Thematic synthesis is a well-recognised method to investigate the acceptability and outcomes of complex interventions [35, 36]. This process involves three distinct steps: line-by-line coding, followed by the generation of descriptive themes, then the development of analytic themes. First, line-by-line coding of text within results sections of identified papers was undertaken independently by TM and EG. Sentences in any way related to the study question were summarily coded with words or phrases as interpreted by the study authors. Initial codes and their meaning, as well as preliminary perspectives of trends or descriptive themes were discussed early among TM and EG and other members of the research team to support reflexivity across the process. As analysis progressed across papers, axial coding was performed to group codes into categories that formed initial descriptive themes. These descriptive themes remained close to the findings of the original text without significant abstraction from the authors. These themes were discussed among TM and EG, with consultation with JJ, whereby more analytic themes that involved researcher interpretation were developed and agreed upon by the authors. These analytic themes involved a higher level of abstraction applied to similar groups of descriptive themes and reflected secondary insights from researchers. These higher-level themes were further divided into descriptive sub-themes to further delineate meaning. Theme one sub-themes could be broadly categorized into micro-, macro- and meso-level mechanisms, contextual levels that have been employed in implementation science and sociology literature [37, 38].

During analysis, trustworthiness was maintained through the use of reflexive journals, data-driven analysis and categorisation of codes, retention of an audit trail for decision-making, and the use of exemplars. The positionality of TM and EG as pharmacists was acknowledged and considered throughout reflexive diaries and consultation with non-pharmacist members of the research team.

### Ethics approval

Not required.

## Results

**Fig. 1:**
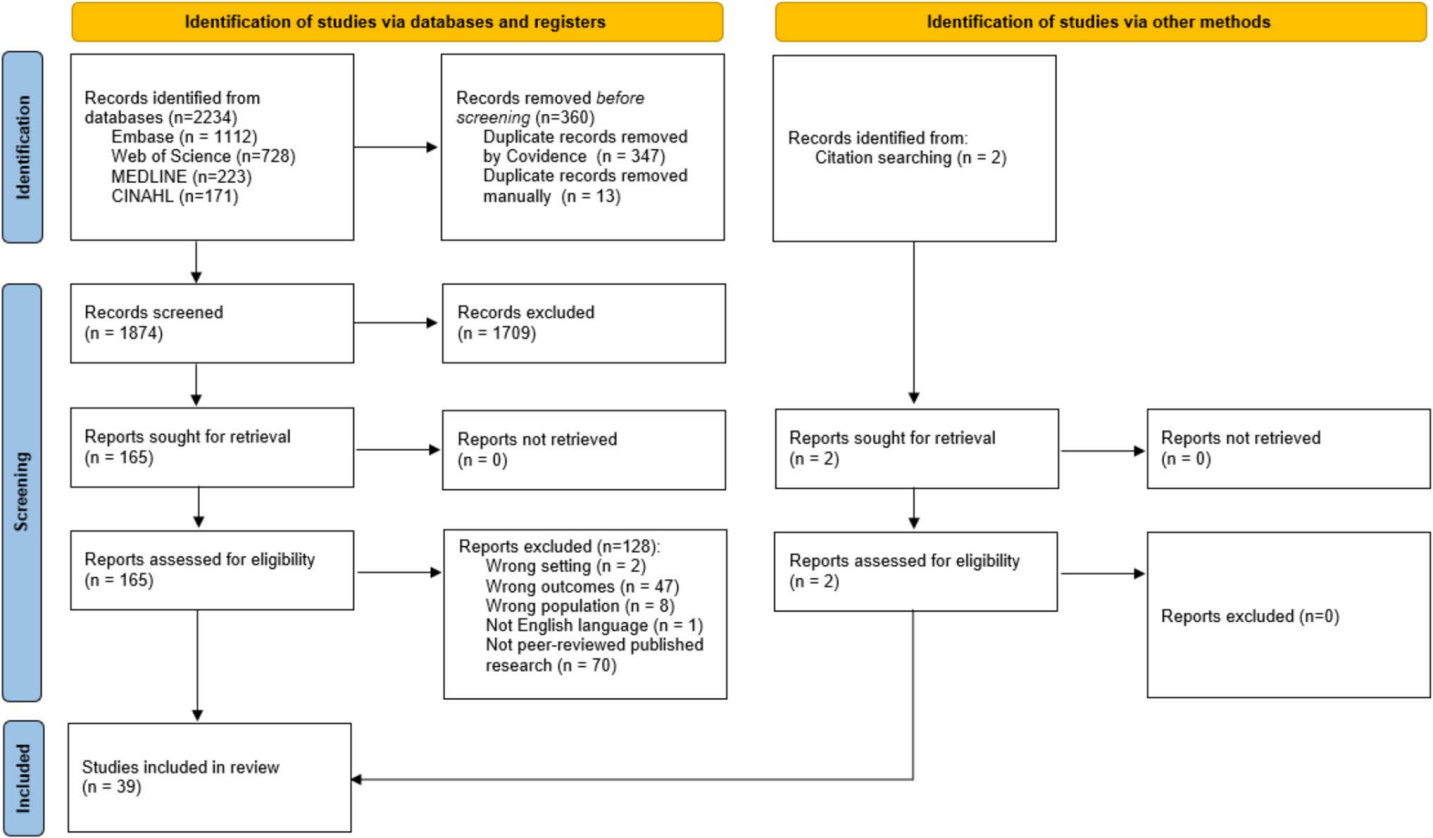
PRISMA 2020 flow-chart of study selection

A total of 2234 records were identified through database searches for initial screening (Fig. 1) with 360 duplicates. The title and abstract screening removed 1709 records resulting in screening 165 full-text papers for eligibility. Of these, there were 37 full text papers that met the inclusion criteria. Citation searching identified two additional papers, resulting in a total of 39 papers for analysis. Critical appraisal of each study is included separately in the Supplementary Information; no papers were excluded based on quality.

### Study characteristics

The 39 included studies were published between 2007 and 2025 (Table 1). Most papers originated from Australia (n = 14, 36%) or the United Kingdom (n = 8, 21%). All but five papers included multiple perspectives from stakeholders: 85% (n = 33) included prescribers, 77% (n = 30) included nurses, and 82% (n = 32) included pharmacists. Other allied health professional perspectives were included in 10% (n = 4) of papers. Most papers (n = 26, 67%) were entirely qualitative in nature, with most of these (n = 24, 92%) using semi-structured interviews alone. Other predominant data collection methods included focus groups (n = 7, 18%) or both focus groups and semi-structured interviews (n = 7, 18%). Thematic analysis as described by Braun and Clarke was the most common qualitative analysis method (n = 16, 41%) [39].

**Table 1:** Study characteristics

Study Characteristics	Participant details (n)	Country	Methodology	Qualitative data collection method	Qualitative analysis method	Objectives	Pharmacist model of care OR role title
Ali et al. [40]	Pharmacists (15)	Australia	Qualitative	Semi-structured interviews	Inductive thematic analysis	Explore views of Australian pharmacists toward reducing the risk of medicines-related harm in residents of RACHs	Residential Medication Management Review, Aged Care Onsite Pharmacist
Anlay et al. [41]	Family physicians (6), physicians with coordinating/consulting roles in RACHs (7), geriatrician (5), pharmacists (6), nurses (4)	Belgium	Qualitative	Semi-structured interviews	Thematic analysis to identify barriers and enablers, followed by linking of barriers and enablers to Theoretical Domains Framework, followed by mapping TDF domains to behavioural change techniques	To explore healthcare professionals’ barriers and enablers for deprescribing and identify theoretical domains for behaviour change to include in future interventions	Visiting model
Anrys et al. [42]	Coordinating physicians, pharmacists, nurse, GPs. [Numbers not reported]	Belgium	Mixed-methods	Focus groups	Thematic analysis	To describe the implementation of a comple cluster-randomised trial to improve prescribing, and explore participant experiences for future implementation	[Novel intervention for research purposes]
Batten et al. [43]	Onsite pharmacists (6), RACH managers (8)	Australia	Mixed-methods	Semi-structured interviews	Framework analysis using a modified checklist	Identification of moderating factors affecting delivery of the onsite pharmacist intervention	Aged Care Onsite Pharmacist
Batten et al. [44]	Residents (10), family members (4), onsite pharmacists (6), prescribers (9), managers (7), and nursing staff (10)	Australia	Mixed-methods	Semi-structured interviews and an adapted survey	Framework analysis using Ritchie and Spencer’s approach [45]	Understand the extent of the normalisation of onsite pharmacists into RACHs	Aged Care Onsite Pharmacist
Batten et al. [31]	Onsite pharmacists (6), RACH managers (7), nurses (10), GPs (7), nurse practitioners (2)	Australia	Mixed-methods	Semi-structured interviews and a survey	Framework analysis [45]	Explore the extent of interprofessional collaboration between onsite pharmacists, prescribers, managers and nursing staff	Aged Care Onsite Pharmacist
Birt et al. [46]	Pharmacist Independent Prescribers (14), GPs (8), care home managers (9), care home staff (6)	England, Scotland and Northern Ireland	Qualitative	Semi-structured interviews	Inductive thematic analysis	To examine stakeholders’ perceptions of how a pharmacist independent prescriber intervention impacted procedures of care homes, as well as resident wellbeing	Pharmacist Independent Prescriber
Birt et al. [47]	Pharmacist Independent Prescribers (16), General Practitioners (6), Care home managers (7)	Scotland, Northern Ireland, England	Mixed-methods	Semi-structured interviews followed by consensus panel	Inductive thematic analysis followed by retroductive approach to map barriers and enablers to the Theoretical Domains Framework, followed by Nominal Group Technique	To examine ongoing Pharmacist Independent Prescriber-led deprescribing following a large-scale trial, develop strategies to address or optimise identified barriers and enablers, and involve stakeholders to develop policy guidance for future behaviour change strategy adoption across United Kingdom care homes	Pharmacist Independent Prescriber
Crunenberg et al. [48]	Community pharmacists (8), coordinating medical physicians (4), GPs (2), nurses (5)	Belgium	Qualitative	Semi-structured interviews	Interpretative phenomenological analysis	Explore the roles of physicians, pharmacists and nurses in interprofessional collaboration, as well as barriers and facilitators to effective collaboration	[Not specified]
Disalvo et al. [49]	Pharmacists accredited to conduct medication reviews (15	Australia	Qualitative	Semi-structured interviews	Inductive coding followed by comparison and allocation of codes to a model for interdisciplinary collaboration [50]	To explore perspectives of pharmacists on residential medication management reviews and the role in improving quality and safety of long-term care prescribing	Residential Medication Management Reviews
Dowd et al. [51]	Geriatrician (3), general practitioner (1), pharmacists (5), nurses (5), physiotherapist (2))	Japan and Australia	Mixed-methods	Semi-structured focus groups via video-conferencing software	Deductive content analysis	Perspectives of healthcare staff on management of chronic non-cancer pain in aged care settings	[Not specified]
Emmerton et al. [52]	Semi-structured interviews: representatives of stakeholder organisations (34) Focus groups: Consumers (25), pharmacists (28), medical practitioners (18)	Australia	Qualitative	Focus groups and semi-structured interviews	Thematic analysis	To identify issues around a theoretical model for pharmacist prescribing in RACF	[Novel intervention for research purposes]
Fleming et al. [53]	General practitioners (10), consultants (4), nurses, pharmacists (9)	Ireland	Qualitative	Semi-structured interviews	Iterative analysis using the Theoretical Domains Framework and behaviour change technique taxonomy	Exploration of healthcare professional views of antibiotic prescribing in long-term care facilities	[Not specified]
Foley et al. [54]	Pharmacists (11), nurses (10) physicians (6)	Switzerland	Qualitative	Focus groups and semi-structured interviews	Content analysis	Perspectives and practice of physicians, nurses and pharmacists on deprescribing	Integrated Pharmacist Services
Gonçalves et al. [55]	Nurses (5), physicians (2), physiotherapist (1), long-term care managers (2), long-term care pharmacists (2), hospital pharmacists (2)	Portugal	Qualitative	Semi-structured interviews	Deductive coding framed by role theory [56], supplemented with further inductive coding	Characterise perception of healthcare professionals and other stakeholders toward pharmacist participation in a national Long-Term Care network	[Not specified]
Halvorsen et al. [57]	Physicians (4), nurses (8)	Norway	Qualitative	Focus groups and in-depth interviews	Systematic text condensation	Exploration of how physicians and nurses experienced collaboration with pharmacists in case conferences	[Not specified]
Haver et al. [58]	Physicians (6), nurses (8), other staff (3)	United States of America	Qualitative	Focus groups	Inductive and deductive thematic analysis	Explore the perceptions of the pharmacist’s impact on the geriatric care team in caring for patients across settings	[Not specified]
Heinrich et al. [59]	GPs (7), pharmacists (7), nurses (12)	Ireland	Qualitative	Semi-structured interviews	Reflexive thematic analysis, then mapped to a framework of deprescribing barriers and enablers, informed by the Theoretical Domains Framework	To identify barriers and enablers to deprescribing in long-term care facilities	[Not specified]
Hill et al. [60]	[Not specified]	Australia	Mixed-methods	Semi-structured interviews	Framework analysis, descriptive statistics	To describe a novel model of care with a pharmacist integrated within a multidisciplinary team, to report medicines-related outcomes for the trial, and describe perspectives of key stakeholders regarding the pharmacist intervention as part of the model of care examined	[Novel intervention of embedded model for research purposes]
Javanparast et al. [61]	Residents (10), family members (2), aged care staff [site manager, registered nurse, enrolled nurse, nurse practitioner] (16), pharmacists [hospital, community, aged care] (19), general practitioners (9), specialist medical consultants (2), persons involved in policy and planning (3)	Australia	Qualitative	Semi-structured interviews	Development of a coding framework using the Consolidated Framework for Implementation Research, followed by thematic analysis	To explore stakeholder perspective on medication management and perceived value of embedding onsite pharmacist in residential aged care homes, and factors that may influence successful implementation of aged care pharmacist at scale	Aged Care Onsite Pharmacist
Kua et al. [62]	Doctors (4), pharmacists (4), nurses (9)	Singapore	Qualitative	Semi-structured interviews	Thematic analysis	To examine the perspectives of healthcare professionals regarding deprescribing knowledge, practice and attitudes	Community-based pharmacists
Kwak et al. [63]	Physicians (4), nurses (3), social workers (5)	Korea	Qualitative	Semi-structured interviews	Inductive thematic analysis	To identify the perspectives of non-pharmacy professionals regarding pharmacist-involved medication management in long-term care facilities	Pharmacist-Involved Medication Management
Laird et al. [64]	Aged care pharmacists (21)	Australia	Qualitative	Semi-structured interviews	Constructivist grounded theory	The perceptions and practices of Australian pharmacists regarding osteoporosis management for aged care residents	Residential Medication Management Reviews
Lane et al. [65]	Pharmacists (25), GPs (24), care home managers (3), care home staff (6), residents (7) and relatives (7)	United Kingdom	Qualitative	Focus groups and semi-structured interviews	Framework analysis using the Theoretical Domains Framework	Stakeholder expectations of what service should be provided by Pharmacist Independent Prescribers (PIPS), what might affect their support for the role, and barriers and enablers to providing the service	Pharmacist Independent Prescribers
Lau et al. [66]	Physicians (4), registered nurses (5), nurse practitioners (2), nursing aid (1), consultant pharmacists (4)	United States of America	Qualitative	Semi-structured interviews	Iterative comparison and evaluation of codes	Exploration of clinical professionals’ perceptions and application of guidelines and how interprofessional interactions affect pharmacotherapy delivery to nursing home residents	Consultant pharmacist
Lim et al. [67]	Nurses (40), GPs (15), pharmacists (6)	Australia	Qualitative	Focus groups and semi-structured interviews	Framework analysis	Exploration of organisational workflow and workplace culture affecting antibiotic prescribing behaviour in residential aged care homes	Residential Medication Management Review
Maidment et al. [68]	Care home managers (5), GPs (3), care staff (13)	United Kingdom	Mixed-methods	Semi-structured interviews	[Not reported]	Expectations and experience of staff before and after an intervention (medication review and health psychology dual intervention for people living with dementia)	[Not specified]
Makan et al. [69]	Elderly Care Specialists (11)	The Netherlands	Qualitative	Focus groups	Open, axial and selective coding	To explore the barriers and enablers of deprescribing among Elderly Care Specialists	[Not specified]
McDerby et al. [70]	GPs (15), Pharmacists (15), nurse (13)	Australia	Mixed-methods	Anonymous surveys with open-text options	Thematic analysis for open-text responses	To analyse perspectives of stakeholders regarding suitability and delivery of the RMMR model for RACH residents living with dementia as well as potential for improvements	Residential Medication Management Review
McGillicuddy et al. [71]	Nurses (18)	Ireland	Qualitative	Semi-structured interviews	Thematic analysis	Investigation of the knowledge, attitudes and beliefs of nursing staff regarding oral medicine modification for older adults	[Not specified]
Mena et al. [72]	Pharmacists, nurses, physicians [numbers not reported]	Switzerland	Mixed-methods	Questionnaires and focus groups	Descriptive statistics and thematic analysis	To evaluate the implementation and impact of the Medication Reviews in Nursing Homes project	Community pharmacists
Meyer-Massetti et al. [73]	Nursing professionals (14), pharmacists (9), physicians (8)	Switzerland	Mixed methods	Survey with free text response options and focus groups	Narrative summary	To survey current medicines use infrastructure and processes, and medication review practices, in RACHs, and explore barriers and facilitators for medication review to become a RACH quality indicator	Visiting model
Palagyi et al. [74]	Residents (25), relatives (16), long-term care facility staff (19), GPs (8), dispensing pharmacists (2), medication review pharmacists (2)	Australia	Qualitative	Focus groups and semi-structured interviews	Thematic analysis using Model of Behaviour Prediction as a framework	Attitudes, norms, self-efficacy, environmental factors, intent, skills and abilities related to perceptions of medication use and deprescribing in LTCFs	Residential Medication Management Reviews
Patterson et al. [75]	Semi-structured interviews: Clinical pharmacists (6) and resident advocates (6). Focus groups: prescribing support pharmacists (14), general practitioners (8), nursing home managers (10)	United Kingdom	Qualitative	Focus groups, semi-structured interviews	Framework analysis using the Fleetwood model [76]	Views and opinions on how the American Fleetwood model may be adapted for use in the UK	Prescribing support pharmacists, clinical pharmacists
Rowe et al. [77]	Physician (1), pharmacist (1), pharmacy student (1), licensed practical nurses (5), residents (3), family members (2)	Canada	Qualitative	Semi-structured interviews	Inductive thematic analysis	The experience and views of physicians, nurses, pharmacists, LTC residents and family members	[Not specified]
Schmüdderich et al. [78]	Registered nurses (14), physicians (3), social workers (2), physiotherapist (1), pharmacist (1), relatives of residents living with dementia (14)	Germany	Mixed-methods	Semi-structured interviews	Deductive-inductive content analysis	Exploration of the current care situation and interprofessional collaboration in the care of residents living with dementia	Visiting model
Sluggett et al. [79]	Interviews: research nurses (4), clinical pharmacist (1), managers or senior registered nurses (8), aged care provider representative (3), residents (3), family member (1), community pharmacist (2), GP (1)	Australia	Mixed-methods	Document analysis (study nurse recruitment record and clinical pharmacist records), semi-structured interviews	Reflexive thematic analysis, informed by phenomenology	The perception of a medicines regimen simplification intervention (SIMPLER intervention)	[Novel medicines simplification intervention for research purposes.]
Visser et al. [80]	Nursing home physicians (13), pharmacists (3)	The Netherlands	Qualitative	Focus groups	Content analysis informed by the Theoretical Domains Framework	To identify barriers and enablers of deprescribing in nursing homes	[Not specified]
Wouters et al. [81]	Residents (6), relatives of residents (8), physicians (8), pharmacists (5), nurses (10)	The Netherlands	Qualitative	Semi-structured interviews	Constant comparative method	Identify barriers and facilitators of conducting medication reviews	[Not specified]

#### Meta-synthesis of data

The meta-synthesis of literature for this systematic review identified three themes:

1. Supporting the role.
2. Medicines expertise activities.
3. Helping the team.

The three themes were perceived to be interlinked. Theme 1 identifies seven sub-themes that describe how the role is supported. These in turn facilitate the content of the role (Theme 2) as described by healthcare professionals. These roles subsequently lead to the outputs of the role as perceived by healthcare professionals (Theme 3). Papers with data supporting themes originated from various geographical settings including the United Kingdom, Australia, United States of America, demonstrating a level of consistency regarding the perceptions of pharmacists working within aged care homes.

### Theme 1: supporting the role

The success of a pharmacist role, as well as its implementation and sustainability, was dependent on the presence or absence of various supports. These could be categorised into micro-, macro- and meso-level factors. Micro-level factors included rapport-building between pharmacists and the wider team, education and experiential background of the pharmacist, access to clinical resources and resident information, and non-technical skills and attributes of pharmacists.

Macro-level factors included the organisational culture and buy-in of key facility stakeholders, as well as clarity of the roles and responsibilities of a pharmacist within the organisation. Meso-level factors included models of care and legislative frameworks that supported implementation and longevity of the pharmacist role.

Across all three levels, interdisciplinary collaboration between pharmacists and other healthcare staff, particularly real-time, in-person collaborative care. When all three levels of factors supported this communication, pharmacists could perform their roles most effectively and healthcare professionals acknowledged their value. Sub-themes are described below in Table 2.

**Table 2:** Theme 1 sub-themes

Sub-theme	Details
Sub-theme 1.1 (Micro-level factor): Building rapport and trust with the inderdisciplinary team supports collaboration and perceived value of pharmacists	Perceived success in the pharmacist’s roles and achievement of outcomes was impacted by the ability to develop trust and relationships with others [42, 43, 46, 49, 58, 60, 66, 70, 74, 75, 78]. When a positive rapport existed between health professionals and pharmacists, this improved perceived efficiency and ‘…*the usefulness of the pharmacist’s role in patient safety was highlighted.”* [48] Trust and rapport were notably built through effective, in-person collaboration with staff and residents, particularly GPs. When pharmacists collaborated in-person, they were “…*on-site and it’s much easier getting together to see the patient and talking through…less misunderstandings and it’s more effective”* [44] [GP perspective] To further develop rapport and trust, healthcare professionals needed to know that pharmacists had considered a resident’s holistic needs, not as a list of medicines and conditions [65, 70]. When staff trusted that the pharmacist had considered the ‘whole’ person, a greater uptake of pharmacist recommendations was noted [65, 70]
Sub-theme 1.2 (Micro-level factor): Education and experiential background enable success and implementation of novel pharmacist roles	Pharmacists identified the need for enhanced educational opportunities and a certain level of experience or clinical background when undertaking the specialised role [40, 42, 43, 53, 55, 61, 63, 64, 72, 80]. Aged care was identified as a distinct area of expertise. If absent, it was identified as a potential barrier to effective collaboration with other health professionals, particularly in countries where there was not a mandatory post-graduate training for pharmacists to work in a RACH setting Undergraduate qualifications and the requisite knowledge required for the specialisation in aged care practice was consistently described as a gap. Dedicated clinical training as well as experience were highlighted as important, with “*mentoring by an experienced pharmacist and residency training in a tertiary care setting…”* [40] identified as a useful method to bridge this gap
Sub-theme 1.3 (Micro-level factor): Access to general resources and residents’ clinical information is integral to the pharmacist role	Pharmacist roles required access to specific resources relevant to practice with older persons, such as the deprescribing tools, anticholinergic burden calculators, interaction checkers, opioid calculators, and more [40, 62, 72, 81, 82] In addition to clinical resources, pharmacists needed access to an individual’s clinical data to inform decision-making. Access improved uptake of recommendations, while a lack of access was seen as prohibitive to effective person-centred recommendations and could impact the quality of the service [41, 60, 64, 66, 67, 70, 72, 73, 75]. Pharmacists required “*the resident’s full clinical picture…necessitating access to both clinical notes and the full medication history*.” [75]
Sub-theme 1.4 (Micro-level factor): Specific attributes of pharmacists are key enablers to success of the role	Attributes commonly associated with success of the pharmacist role included flexibility, proactivity, and communication skills [31, 43, 48, 49, 54, 55, 60, 62, 65, 66, 81] Pharmacists needed to be flexible to the views and priorities of other team members and residents. It was important for pharmacists to consider the ‘whole’ person and provide holistic recommendations rather than simply following guidelines [54, 60, 62, 66, 81] Communication skills and proactivity were key enablers in developing relationships in addition to supporting the uptake of clinical recommendations [31, 43, 48, 49, 55, 55]
Sub-theme 1.5 (Macro-level factor): Supportive organisations and buy-in enables successful implementation of the role	The organisational culture and buy-in of management personnel were reported as factors for successfully implementing new pharmacist services in aged care. Organisations that prioritised and acknowledged the importance of collaborative working relationships were seen to optimally leverage the pharmacist role and its associated impact [43, 48, 61] This could be seen through simple interventions such as ensuring ease of access and visibility of the pharmacist when onsite. This perceived buy-in from the organisation and a “*positive culture focussed on collaboration*” [43] had flow-on effects and supported and motivated pharmacists to deliver collaborative care to individuals
Sub-theme 1.6 (Meso-level factor): Favourable models of care enable successful implementation and impact of the role	Models of care wherein pharmacists were consistently and physically available to staff were considered advantageous to the integration and impact of the pharmacist role. Perceived advantages to models of care that featured a consistent onsite pharmacist were described across countries where this model was available [44, 46, 49, 64, 70] Models in which the pharmacist was an infrequent external visitor, as opposed to regularly present as a team member, were associated with barriers affecting integration and provision of care [40, 49, 64, 67, 74, 77]. Many stakeholders perceived that a visiting model was not fit for purpose as it “*provides episodic limited care*” [64]. Stakeholders further “*felt negatively*” [48] with the increased workload related to these roles, with nursing staff acting as intermediaries between prescribers and pharmacists. Periodic medication reviews were at times described as being of low quality, with stakeholders referring to ‘copy-and-paste’ reports that were not useful; this issue was not described in models of care involving integrated roles [40, 49]
Sub-theme 1.7 (Macro-level factor): Role clarity of a pharmacist enables successful integration into existing teams	Roles and responsibilities that were clear to stakeholders were considered enablers of implementation of roles and collaboration [42, 43, 46, 49, 52, 61, 65]. Health professionals identified a need to be made aware of the purpose and content of the pharmacist role, particularly when newly implemented [46, 70, 79]. Instructive role descriptions were useful guidance for onsite pharmacists and managers when integrating new roles [43, 61, 65]

### Theme 2: medicines expertise activities

Clear activities of value to healthcare professionals were identified throughout the papers, included below as perceived by these professionals. These activities revolved around a perception of a pharmacist’s medicines expertise. These could be characterised into three overarching descriptive categories that were perceived to support synergy with existing professionals and roles. The three categories described in Table 3 include ‘knowledge and communication brokers’, ‘filling existing gaps in care’, and ‘quality use of medicines activities’. Pharmacists were broadly considered to be a useful complement to existing teams through either introducing new and specific medicines-related activities or taking responsibility for existing activities.

**Table 3:** Theme 2 sub-themes

Sub-theme	Details
Sub-theme 2.1: Pharmacists are knowledge and communication brokers for staff, residents and caregivers	A key pharmacist role was their input as a knowledge and communication broker. Pharmacists had a key role in delivering education to staff, residents, and family members, and this was expressed by GPs, RACH managers, nursing staff and pharmacists themselves [40, 48, 55, 58, 60, 61, 63–65, 68, 71, 79] Pharmacists were considered to improve communication within facilities, where they optimised and facilitated communication between GPs and other healthcare staff, staff and residents, and staff and families [31, 44, 54, 60, 63, 65, 81] This was not universal, however. Nurses interacting with visiting pharmacists found that they took on communication broker roles between GPs and pharmacists and “*nurses perceived this…as a resource and time-consuming role*” [48]
Sub-theme 2.2: Pharmacists fill existing gaps in care to support the wider interdisciplinary team	Pharmacists were frequently described as contributing to fill gaps in existing models of care, particularly in the context of significant workloads of other staff. The intricacies of medicines management were described as an afterthought by prescribers and nurses and pharmacists could support this [44, 57, 62, 65, 69, 77]. GPs particularly believed that pharmacists provided necessary supports to improve medicines use, particularly helping to “*tie up all those loose ends”* [65]
Sub-theme 2.3: Pharmacists play a pivotal role in optimising quality use of medicines	Pharmacists contributed to person-centred, quality use of medicines, both broadly and through specific activities [31, 40, 42–44, 46, 48, 51–53, 55, 57, 63–65, 68, 70]. Particularly when pharmacists were embedded within facilities as a team member, medicines management at a facility level was believed to improve [43, 51–53, 65] Specific roles pertaining to medicines management for pharmacists included participating in case conferences [42, 57], screening for interactions [53, 63], developing and implementing guidelines [66, 71], leading and assisting with deprescribing interventions [44, 64, 70, 77], medicines reconciliation [44, 63], optimising transitions of care [55], and assisting with modification of oral medicines [71] Many of these useful roles were facilitated by having the pharmacist onsite, particularly with case conferences where “*the preparation made, mainly by pharmacists, was considered an advantage*” [42]

### Theme 3: helping the team

Healthcare professionals recognised a range of outputs of the pharmacist role that pivoted around *helping the team*. Perceived outputs were stratified into three sub-themes all related to contributions to the aged care team. These included confidence of staff and residents, enhanced staff efficiency, and improved person-centred care. Pharmacists had the potential to enhance self-perceived confidence of their healthcare colleagues related to medicines management, individual and team efficiency, and ultimately were believed to improve the quality of care to residents.

Outcomes of pharmacist roles were seen across jurisdictions and models of care, but were most pronounced when collaboration between professionals was optimised. Outcomes are described as complementary to existing healthcare professional roles (Table 4).

**Table 4:** Theme 3 sub-themes

Sub-theme	Detail
Sub-theme 3.1: Pharmacists can enhance confidence of staff, residents and residents’ families	Nursing staff, managers, and GPs felt an improved sense of confidence and reassurance in their own roles as well as medicines safety across the facility [31, 46, 55, 58, 80]. Prescribers particularly identified that pharmacists supported their role in prescribing or deprescribing, and that they offered “*‘another pair of eyes’ to increase patient safety…*” [46]. Prescribers felt that pharmacists increased their ability to “*deprescribe with confidence*” [80] The ability of pharmacists to directly interact with and empower residents and their families was seen to be of further value. Staff described potential or actual improved confidence and reassurance among residents and their families who could seek clarification around medicines, particularly regarding changes [44, 58, 65]. This was noted specifically when the pharmacist was in concordance with a prescriber’s decisions [81]
Sub-theme 3.2: Pharmacists improve capacity and capability of the workforce	Pharmacists were described as improving efficiency in individual tasks as well as services more generally. This benefit to workload included a range of staff, including managers, GPs and nursing staff [31, 44, 46, 58, 60, 61, 69] This improved capacity and capability was described at times as introducing new activities to the existing care models, such as medicines reviews, and at times as taking on existing medicines-related activities that better suited their skillset, such as deprescribing or auditing of medicines use [44, 51, 63, 70, 77] GPs identified that pharmacists complemented their roles in diverse ways, often streamlining activities such as deprescribing, medication reviews, and liaising with specialists [44, 46, 61, 79, 81] However, this was not universal, as some staff reported an increased workload due to greater scrutiny of medications, with one GP noting that the pharmacist would review *“a lot of the medication, a lot more than I would””* [GP perspective] [44]
Sub-theme 3.3: Pharmacists can improve person-centred care	Pharmacists were particularly valued when they spent time assessing individual residents holistically, considering individual needs, and enhancing overall medicines management [44, 46, 52, 53, 55, 57, 60, 65, 70, 75] Ultimately, healthcare professionals perceived that the pharmacist was able to improve the provision of person-centred care within RACHs. This improved care was perceived on an individual level [44, 55, 57, 70, 75], as well as a facility [43, 51–53, 65] and even health system level [60]

## Discussion

This paper has summarised existing qualitative evidence pertaining to pharmacist roles in aged care. We have described in this paper the perceived enablers that support pharmacist roles in aged care, pharmacist roles that are valued by team members, and the key outcomes of these roles as perceived by healthcare professionals. An array of stakeholders, particularly general practitioners and nursing managers, describe a range of benefits of pharmacist services to the provision of healthcare for older persons residing in RACHs, complementing existing teams in diverse and meaningful ways. Pharmacist roles that leverage, prioritise and support existing interdisciplinary collaboration within healthcare teams are most valued. These interdisciplinary roles are perceived to improve person-centred, collaborative healthcare provision to older persons in residential aged care.

The roles of pharmacists most valued by staff were clear throughout the papers, complementing the existing teams and skillsets. Pharmacists either introduced new medicines-specific activities such as medicines reviews, or took control of existing activities suitable to their expertise, such as leading deprescribing or monitoring and enforcing guideline compliance. The roles could be categorised across three domains, described as sub-themes. Pharmacists acted as knowledge brokers and facilitators of communication; this is a recognised role for health professionals when translating novel interventions and practices into health settings [83–85]. These broker roles offer solutions to the well-documented issues associated with transitions of care and medicines discrepancies arising at these times. This aligns with existing recommendations for aged care homes [86–88]. Participants also believed that pharmacists could fill existing gaps in care. This is particularly pertinent given the high workloads and complexities inherent in aged care, as well as the increasing pressures on health services for older adults [4]. Additionally, health professionals identified that pharmacists optimised the quality use of medicines through distinct activities, including deprescribing, screening for interactions, and medicines reconciliation. These are essential tasks in aged care, where people are more likely to be exposed to polypharmacy, complaints to services are most commonly medicines-related, and medication errors are frequent [89–92].

This work has also identified a range of supports that assist pharmacists to undertake services within aged care homes, particularly when these are novel roles for the home or the pharmacist. Dedicated training and the benefits of experience were observed to support pharmacists, with specific reference made to ‘*mentoring by an experienced pharmacist and residency training*’ [40] as useful to provide necessary experience. This aligns with existing research on the preparedness of pharmacists to work in embedded aged care roles, which has identified the need for both further education and experience [93].

Clear roles and responsibilities for pharmacists were important for success. Role ambiguity as a barrier to integrating novel healthcare services is prevalent in the literature, and has emerged in similar work in the implementation of novel pharmacist services and other interventions in the hospital setting [94–97]. In this existing research, success in novel services is often dependent on clear accountability of roles and responsibilities [94–97].

Pharmacists not embedded within the team were associated with a range of perceived barriers. Embedded roles were able to foster communication and develop trust and rapport, supporting the uptake of pharmacist recommendations. In these roles, pharmacists were able to improve communication and knowledge brokerage within existing teams, as well as reduce time-consuming roles of nursing and other staff. In contrast, visiting pharmacist roles required nursing staff to broker communication resulting in an increased workload and negative perceptions of the role [48]. Visiting pharmacist roles were associated with criticisms regarding quality of medicine review reports, which was not expressed in response to more integrated roles. Other research has found significant variability in content of visiting pharmacist reports and uptake of recommendations [98–100]. Truly interdisciplinary healthcare delivery is vital to health services and is positively associated with person-centred outcomes [10, 14, 101]. A 2021 systematic review on barriers and enablers to pharmacist integration in ward-based interdisciplinary teams found similar results [102]. In that paper, knowledge and skills, interpersonal relationships, and co-location with other professionals enabled integration into teams [102]. Additional research on pharmacists integrating into ward-based teams identified that the development of collaborative working relationships further depends upon actively using communication and other skills to holistically improve person-centred care [30]. Our paper reflects this and identifies further enablers that may assist future development of models of care and implementation of novel pharmacist services in the aged care setting.

Pharmacists were perceived to achieve specific, valuable outcomes for individual healthcare professionals, healthcare teams, residents, and families. Enhanced confidence of stakeholders was often described. Low confidence of healthcare staff is a predictor of burnout and is a particularly relevant consideration in a sector where workforce retention remains a concern [103, 104]. Pharmacists were also described as improving efficiency and capacity of the healthcare team, which was identified as an important outcome in a health service environment marked by increasing pressures on systems and workforces [4]. Finally, staff believed that pharmacists were able to improve medicines management at an individual, facility and health system level. The value that healthcare professionals place on these perceived outcomes should instil confidence for policymakers and organisations looking to implement such services.

The themes identified in this work may assist policymakers, organisations and healthcare teams to optimise current and future implementation of pharmacist services into aged care homes. Policymakers introducing or modifying pharmacist models of care may benefit from ensuring that such models facilitate interdisciplinary collaboration. Organisations implementing pharmacist services into aged care homes may benefit from ensuring clarity of the role, supporting education and mentorship, fostering integration into existing teams, and ensuring access to information and resources. Pharmacists assuming aged care roles may benefit from targeted efforts to build rapport and trust with other staff, and pursue opportunities to develop particular clinical and non-clinical skills and attributes.

### Strengths and limitations

This study is limited by the range of stakeholder perspectives, largely composed of nursing and medical staff, and lack of allied health and consumer perspectives. It is further limited by the method; triangulation of qualitative data through the analysis of quantitative survey data may strengthen findings. As grey literature was not searched, selection bias for only published literature is also a limitation. As English language only was included in the search, this may have missed key perspectives from international literature not published in English. The paper is strengthened by the inclusion of international literature and identification of consistencies and trends across geographical locations and models of care.

### Future research

Future primary research may benefit from gathering more diverse perspectives from allied health professionals, as perspectives within the identified papers primarily involved nursing staff, prescribers, and pharmacists. This is particularly important given the crossover between allied health professionals and pharmacists and potential for collaboration (such as speech pathologists or physiotherapists, managing swallowing difficulties and pain respectively). Investigations on improving interdisciplinary collaboration with visiting pharmacist models of care should also be undertaken. Future literature reviews should investigate the perspectives of residents, informal caregivers, and non-health professional staff to complement these findings. Although we did not evaluate the outcomes of pharmacist roles in aged care, this has been explored previously with mixed findings in aged care specifically [24] and care of older persons broadly [105]; future research could employ methodologies such as realist evaluation to identify why and in what contexts such outcomes occur.

## Conclusion

This synthesis of the current evidence regarding the perceptions of healthcare professionals on the role of pharmacists in aged care provides background information for individuals, organisations and health policy developers for future implementation. Synthesis of the available evidence indicates that pharmacists are believed to improve stakeholder confidence, staff capacity and capability, and overall person-centred care for persons residing in aged care. Embedded roles that foster interdisciplinary collaboration are preferred to irregular visiting roles. These embedded roles are enabled through a range of mechanisms that policymakers, organisations and individuals may leverage for successful implementation in future iterations.

## Supplementary Information

Below is the link to the electronic supplementary material.

Supplementary file1 (XLSX 12 KB)

Supplementary file2 (DOCX 21 KB)

## Data Availability

Data are available from the corresponding author upon reasonable request.
