# Supplementary material for: Healthcare professionals’ perspectives of pharmacist roles in residential aged care: a qualitative systematic review and meta-synthesis
Source: Int J Clin Pharm. 2026 May 28;48(4):1246–66. doi: 10.1007/s11096-026-02166-4 (PMC13368974; doi:10.1007/s11096-026-02166-4)
Supplement: Supplementary file 2 — Supplementary file2 (DOCX 21 KB) [file 11096_2026_2166_MOESM2_ESM.docx]

**Supplementary Table 1: Database Search Strategy**

| **Database** | **Search strategy** |
| --- | --- |
| **Embase** | 1. Exp attitude 2. Exp attitude of health personnel 3. Exp health knowledge, attitudes, practice 4. Attitude*.ti,ab,kw 5. Exp perception 6. Belief*.ti,ab,kw 7. Perspective*.ti,ab,kw 8. Expectation*.ti,ab,kw 9. Experience*.ti,ab,kw 10. Preference*.ti,ab,kw 11. View*.ti,ab,kw 12. Desire*.ti,ab,kw 13. Assess*.ti,ab,kw 14. Aged Care Facility.ti,ab,kw 15. Home* for the aged.ti,ab,kw 16. Exp nursing homes 17. Nursing facility.ti,ab,kw 18. Exp Homes for the aged 19. long-term care facilit*.ti,ab,kw 20. Pharmac*.ti,ab,kw 21. Exp pharmacy service 22. Exp pharmacist 23. Medical.ti,ab,kw 24. Exp physicians 25. Exp medical staff 26. Doctor*.ti,ab,kw 27. Allied health.ti,ab,kw 28. Exp allied health occupations 29. Exp allied health personnel 30. Podiatr*.ti,ab,kw 31. Exp podiatry 32. Physiotherap*.ti,ab,kw 33. Speech patholog*.ti,ab,kw 34. Exp speech-language pathology 35. Exp Physical therapists 36. Exercise physiolog*.ti,ab,kw 37. Social work*.ti,ab,kw 38. Exp social work 39. Optometr*.ti,ab,kw 40. Dentist*.ti,ab,kw 41. Occupational therap*.ti,ab,kw 42. Psycholog*.ti,ab,kw 43. Dietitian*.ti,ab,kw 44. Exp nurses 45. nurs*.ti,ab,kw 46. OR/ 1-13 47. OR/ 14-19 48. OR/ 20-22 49. OR/ 23-43 50. 44 AND 45 AND 46 AND 47 51. Limit 50 to English language only 52. Limit 51 to publications published after 2000 |
| **Medline** | 1. Exp attitude 2. Exp attitude of health personnel 3. Exp health knowledge, attitudes, practice 4. Attitude* 5. Exp perception 6. Belief* 7. Perspective* 8. Expectation* 9. Experience* 10. Preference* 11. View* 12. Desire* 13. Assess* 14. Aged Care Facility 15. Home* for the aged 16. Exp nursing homes 17. Nursing facility 18. Exp Homes for the aged 19. long-term care facilit* 20. Exp home nursing 21. Aged care 22. Exp Health Services for the Aged 23. Exp Homes for the aged 24. Exp Nursing homes 25. Home* for the aged 26. Exp long-term care 27. Long-term care facilities 28. Pharmacy 29. Pharmacist* 30. Exp pharmacists 31. Medical 32. Exp physicians 33. Exp medical staff 34. Doctor* 35. Allied health 36. Exp allied health occupations 37. Exp allied health personnel 38. Podiatr* 39. Exp podiatry 40. Physiotherap* 41. Speech patholog* 42. Exp speech-language pathology 43. Exp Physical therapists 44. Exercise physiolog* 45. Social work* 46. Exp social work 47. exp Optometry/ 48. optometr*.mp. 49. exp Optometrists/ 50. dentist*.mp. 51. exp Dentists/ 52. occupational therapist.mp. 53. exp Occupational Therapists/ 54. psychologist*.mp 55. exp psychology/ 56. dietitian*.mp 57. exp dietitian 58. Exp nurses 59. nurs* 60. OR/ 1-13 61. OR/ 20-27 62. OR/ 28-30 63. OR/ 31-59 64. 60 AND 61 AND 62 AND 63 65. Limit 64 to English language only 66. Limit 65 to publications published after 2000. |
| **CINAHL** | 1. (MH Attitude+) 2. attitude* 3. (MH "Attitude of Health Personnel+") 4. (MH "Health Knowledge, Attitudes, Practice+") 5. (attitude* OR belief* OR feedback OR perspective* OR opinion* OR expectation* OR preference* OR view* OR desire*) 6. (MH "Home Nursing+") 7. "aged care" 8. (MH "Health Services for the Aged+") 9. (MH "Homes for the Aged+") 10. (MH "Nursing Homes+") 11. "home* for the aged" 12. (MH "Long-Term Care+") 13. "long-term care facilities" 14. (MH Pharmacists+) 15. Pharmacy or pharmacist 16. (MH Physicians+) 17. (MH "Medical Staff+") 18. (MH "Medical Staff+") 19. doctor* 20. (MH "Allied Health Occupations+") 21. (MH "Allied Health Personnel+") 22. (MH Podiatry+) 23. podiatr* 24. (MH "Physical Therapists+") 25. physiotherap* 26. (MH "Social Work+") 27. "social work*" 28. (MH Nurses+) 29. nurs* 30. "exercise physiolog*" 31. (MH "Speech-Language Pathology+") 32. "allied health*" 33. OR/ 1-5 34. OR/ 6-13 35. OR/ 14, 15 36. OR/ 16-32 37. 33 AND 34 AND 35 AND 36 38. Limit 37 to English language only 39. Limit 38 to publications published after 2000. |
| **Web of Science** | 1. "Exp attitude" 2. "Exp attitude of health personnel" 3. "Exp health knowledge, attitudes, practice" 4. Attitude* 5. "Exp perception" 6. Belief* 7. Perspective* 8. Expectation* 9. Experience 10. Preference* 11. View* 12. Desire* 13. Assess* 14. "Aged Care Facility" 15. "Home* for the aged" 16. "Exp nursing homes" 17. "Nursing facility" 18. "Exp Homes for the aged" 19. "long-term care facilit*" 20. "Exp home nursing" 21. "Aged care" 22. "Exp Health Services for the Aged" 23. "Exp Homes for the aged" 24. "Exp Nursing homes” 25. "Home* for the aged" 26. "Exp long-term care" 27. "Long-term care facilities" 28. Nursing-home resident 29. Pharmacy 30. pharmacist* 31. "Exp pharmacists" 32. Medical 33. "Exp physicians" 34. "Exp medical staff" 35. Doctor* 36. "Allied health" 37. "Exp allied health occupations" 38. "Exp allied health personnel" 39. Podiatr* 40. "Exp podiatry" 41. Physiotherap* 42. "Speech patholog*" 43. "Exp speech-language pathology" 44. "Exp Physical therapists" 45. "Exercise physiolog*" 46. "Social work*" 47. "social work 48. exp Optometry" 49. optometr* 50. Optometrists 51. dentist* 52. Dentists 53. "occupational therapist" 54. "Occupational Therapists" 55. psychologist* 56. psychology 57. dietitian* 58. "exp dietitian" 59. "Exp nurses" 60. nurs* 61. OR/ 1-13 62. OR/ 14-28 63. OR/ 29-31 64. OR/ 32-60 65. AND/ 61-64 66. Limit 65 to English language only 67. Limit 66 to publications published after 2000. |
